# Graves’ disease after exposure to the SARS-CoV-2 vaccine: a case report and review of the literature

**DOI:** 10.1186/s12902-023-01387-2

**Published:** 2023-06-15

**Authors:** Kai Takedani, Masakazu Notsu, Naoto Ishiai, Yu Asami, Kazuhiko Uchida, Keizo Kanasaki

**Affiliations:** 1 Department of Internal Medicine, Masuda Red Cross Hospital, Masuda, Shimane Japan; 2 Department of Internal Medicine 1, Faculty of Medicine, Shimane University, 89-1 Enya-Cho, Izumo, Shimane 693-8501 Japan; 3 Department of Cardiology, Masuda Red Cross Hospital, Masuda, Shimane Japan

**Keywords:** Graves’ disease, COVID-19, Vaccine, Autoimmune/inflammatory syndrome induced by adjuvants

## Abstract

**Background:**

Autoimmune/inflammatory syndrome induced by adjuvants (ASIA) is characterized by immune system dysregulation after exposure to adjuvants, such as aluminum. Although cases of autoimmune thyroid diseases caused by ASIA have been reported, Graves' disease is one of the rarer diseases. There are some reports that vaccines against severe acute respiratory syndrome coronavirus 2 (SARS-CoV-2) cause ASIA. Here, we describe a case of Graves’ disease following SARS-CoV-2 vaccination and a review of the literature.

**Case presentation:**

A 41-year-old woman was admitted to our hospital because of palpitations and fatigue. Two weeks after receiving the second SARS-CoV-2 vaccine (BNT162b2, Coronavirus Modified Uridine messenger RNA (mRNA) Vaccine, Pfizer), she developed fatigue and gradually worsened. On admission, she exhibited thyrotoxicosis (thyroid-stimulating hormone (TSH) < 0.01 mIU/L (0.08–0.54), free triiodothyronine (FT3) 33.2 pmol/L (3.8–6.3), and free thyroxine (FT4) 72.1 pmol/L (11.6–19.3)) and palpitations associated with atrial fibrillation. TSH receptor antibody (TRAb) was positive (TRAb 5.0 IU/L (< 2.0)), and ^99m^Tc scintigraphy showed diffuse uptake in the thyroid gland, suggesting that the thyrotoxicosis in this case was caused by Graves’ disease. Thiamazole was prescribed to correct her condition, and soon after this treatment was initiated, her symptoms and thyroid hormone levels were significantly reduced.

**Conclusions:**

This case report reinforces the potential correlation between ASIA affecting the thyroid and SARS-CoV-2 mRNA vaccines. The clinical course suggests that it is essential to consider the possibility of developing ASIA, such as Graves' disease, after exposure to the SARS-CoV-2 vaccine.

## Background

Graves’ disease is an autoimmune thyroid disease (AITD) and is recognized by the presence of thyroid-stimulating hormone (TSH) receptor antibody (TRAb) [1, 2]. That constitutively activates the thyroid gland, leading to a state of diffuse hyperthyroidism. Graves’ disease is the most common cause of hyperthyroidism. The underlying factors involved in the development of Graves' disease remain uncertain. A few genetic loci have been suggested, as well as smoking, stress, and exposure to great amounts of iodine, despite a lack of conclusive causal relationships [3].

Adjuvants encompass several substances commonly used in vaccines to boost immune reactivity toward antigens, and autoimmune/inflammatory syndrome induced by adjuvants (ASIA) is characterized by innate and adaptive immune system dysregulation after exposure to adjuvants, such as aluminum [4, 5]. Although cases of AITD caused by ASIA have been reported, most of them were destructive thyroiditis, and Graves' disease is a rare occurrence [6–8]. COVID-19 poses a significant threat to the entire world. Vaccines of different technologies and types have been developed to reduce COVID-19 infection and severity [9]. However, there are some reports that vaccines against severe acute respiratory syndrome coronavirus 2 (SARS-CoV-2) cause ASIA, including cases of Graves’ disease [10, 11].

Here, we present a case of Graves’ disease following SARS-CoV-2 vaccination and describe other cases found through a literature review.

## Case presentation

A 41-year-old woman was admitted to our hospital because of palpitations and fatigue. Three months prior to hospitalization, she received the first vaccine for SARS-CoV-2 (BNT162b2, Coronavirus Modified Uridine messenger RNA (mRNA) Vaccine, Pfizer), and only mild pain at the vaccination site was observed. Three weeks later, she received the second dose of the vaccine. The next day, she had a fever and took acetaminophen orally, and the fever disappeared in one day. Two weeks later, she developed fatigue and gradually worsened. A week before admission, she went to the emergency department because of palpitations associated with atrial fibrillation and demonstrated thyrotoxicosis (TSH < 0.01 mIU/L (0.08–0.54, CLEIA method), free triiodothyronine (FT3) 28.3 pmol/L (3.8–6.3, CLEIA method), and free thyroxine (FT4) 61.8 pmol/L (11.6–19.3, CLEIA method)). At that time, she went home having received only symptomatic treatment. At the follow-up visit, her general status was not improved, and she was hospitalized to treat thyrotoxicosis.

She had no family history of endocrine diseases, including thyroid disease. She was a nonsmoker and nondrinker. She was not exposed to intense stress or excess iodine. She had no past history and did not have a preceding cold. She did not have a prior COVID-19 infection. Two months before the appearance of her symptoms, the electrocardiogram showed sinus rhythm, and 18 months before, her thyroid hormone levels were normal. Her body weight had decreased by 3 kg in the three months before admission. Her menstruation was regular before hospitalization, and she was not pregnant. She had irregularly taken iron supplements due to iron deficiency anemia.

Her height was 163 cm, her body weight was 39 kg, and her body mass index (BMI) was 14.7 kg/m^2^. Her consciousness was clear. Her blood pressure was 107/52 mmHg, her pulse rate was 100 beats/min and irregular, her body temperature was 36.7 °C, and her oxygen saturation (SpO_2_) was 100%. Her anterior neck was not swollen and without pain. She had a tremor. She had diarrhea without stomach pain. The other physical findings were unremarkable. She did not have eye symptoms.

**Fig. 1:**
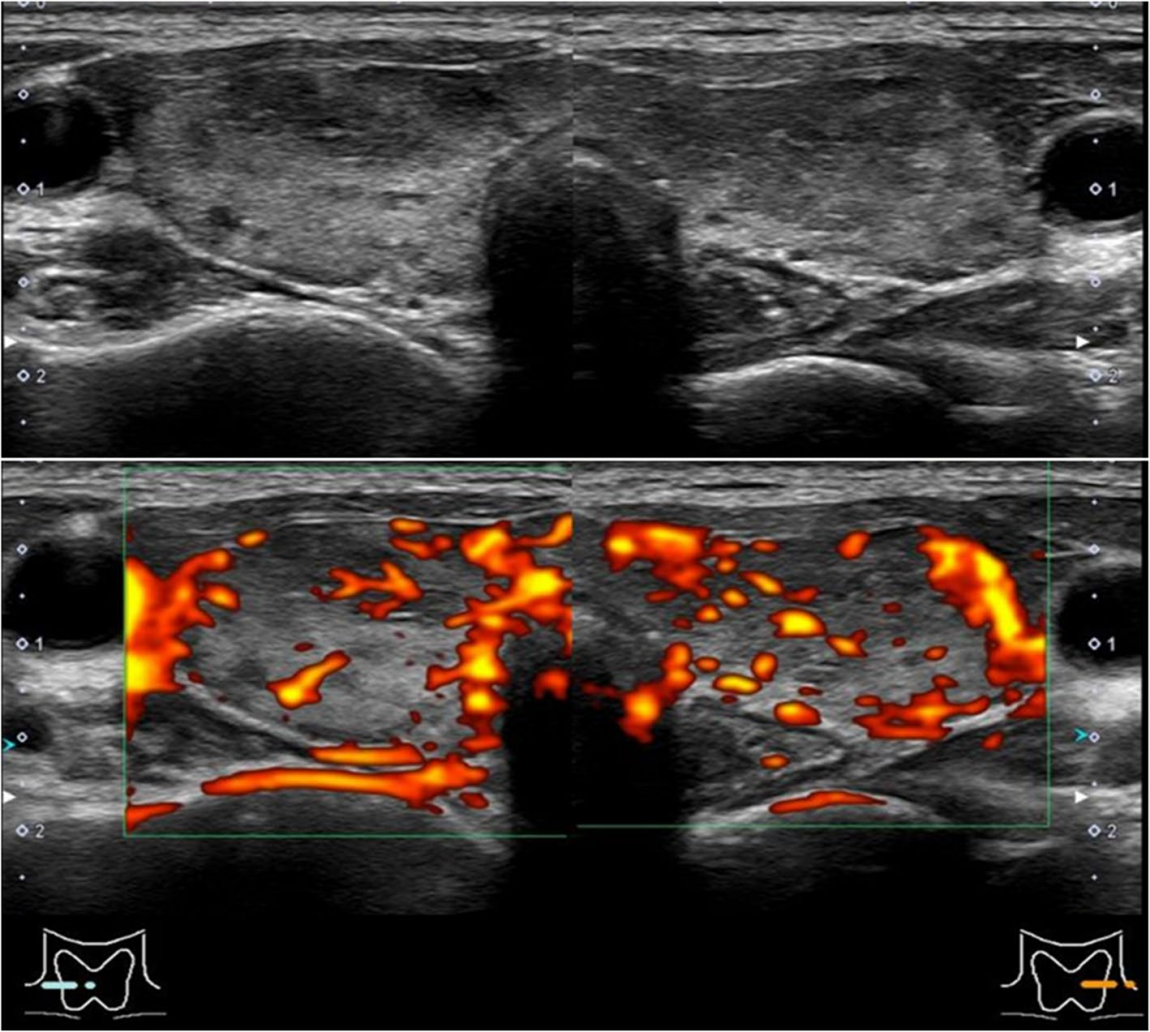
Thyroid ultrasonography. Right lobe: 15 × 50 × 13 mm. Left lobe: 13 × 50 × 11 mm. Increased blood flow throughout the thyroid gland without diffuse goiter was revealed

**Fig. 2:**
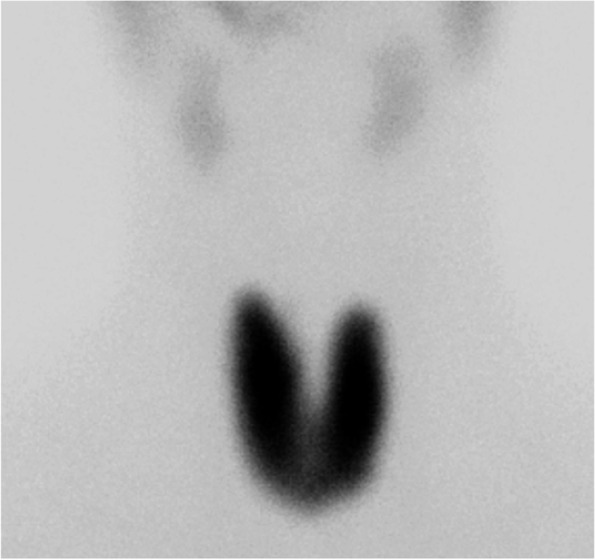
^99m^Tc scintigraphy. Diffuse uptake in the thyroid gland was revealed

The laboratory findings on admission are shown in Table 1. She had severe thyrotoxicosis (TSH < 0.01 mIU/L, FT3 33.2 pmol/L, FT4 72.1 pmol/L). The presence of only gastrointestinal manifestation (diarrhea) suggested that she did not have a thyroid crisis. TRAb and thyroid-stimulating antibody (TSAb) were positive (TRAb 5.0 IU/L (< 2.0, CLEIA method) and TSAb 262% (< 120, EIA method)). Inflammatory markers were not elevated. Thyroglobulin antibody (Tg-Ab) and thyroid peroxidase antibody (TPO-Ab) were negative. Antinuclear antibody, which was screened to check for other autoimmune diseases, was also negative. Ultrasound revealed heterogeneous tissue and increased blood flow throughout the thyroid gland without diffuse goiter (Fig. 1). ^99m^Tc pertechnetate scintigraphy showed diffuse uptake in the thyroid gland, and ^99m^Tc uptake was 11.0% (0.5–3.5) (Fig. 2). These findings suggested that thyrotoxicosis in this case was caused by Graves’ disease, not destructive thyroiditis. An electrocardiogram showed slight tachycardia due to atrial fibrillation. Echocardiography revealed no abnormalities.

**Table 1:** Baseline laboratory data

Parameter	Observed	Reference range
Serum characteristics		
CRP, mg/dL	0.1	< 0.3
ESR (1 h), mm	13	3–15
FT3, pmol/L	33.2	3.8–6.3
FT4, pmol/L	72.1	11.6–19.3
TSH, mIU/L	< 0.01	0.08–0.54
TRAb, IU/L	5.0	< 2.0
TSAb, %	262	< 120
Tg-Ab, IU/mL	0.9	< 4.1
TPO-Ab, IU/mL	2.2	< 5.6
Tg, ng/mL	121	< 34

*CRP* C-reactive protein, *ESR* erythrocyte sedimentation rate, *FT3* free triiodothyronine, *FT4* free thyroxine, *TSH* thyroid-stimulating hormone, *TRAb* TSH receptor antibody, *TSAb* thyroid stimulating antibody, *Tg-Ab* thyroglobulin antibody, *TPO-Ab* thyroid peroxidase antibody, *Tg* thyroglobulin

**Fig. 3:**
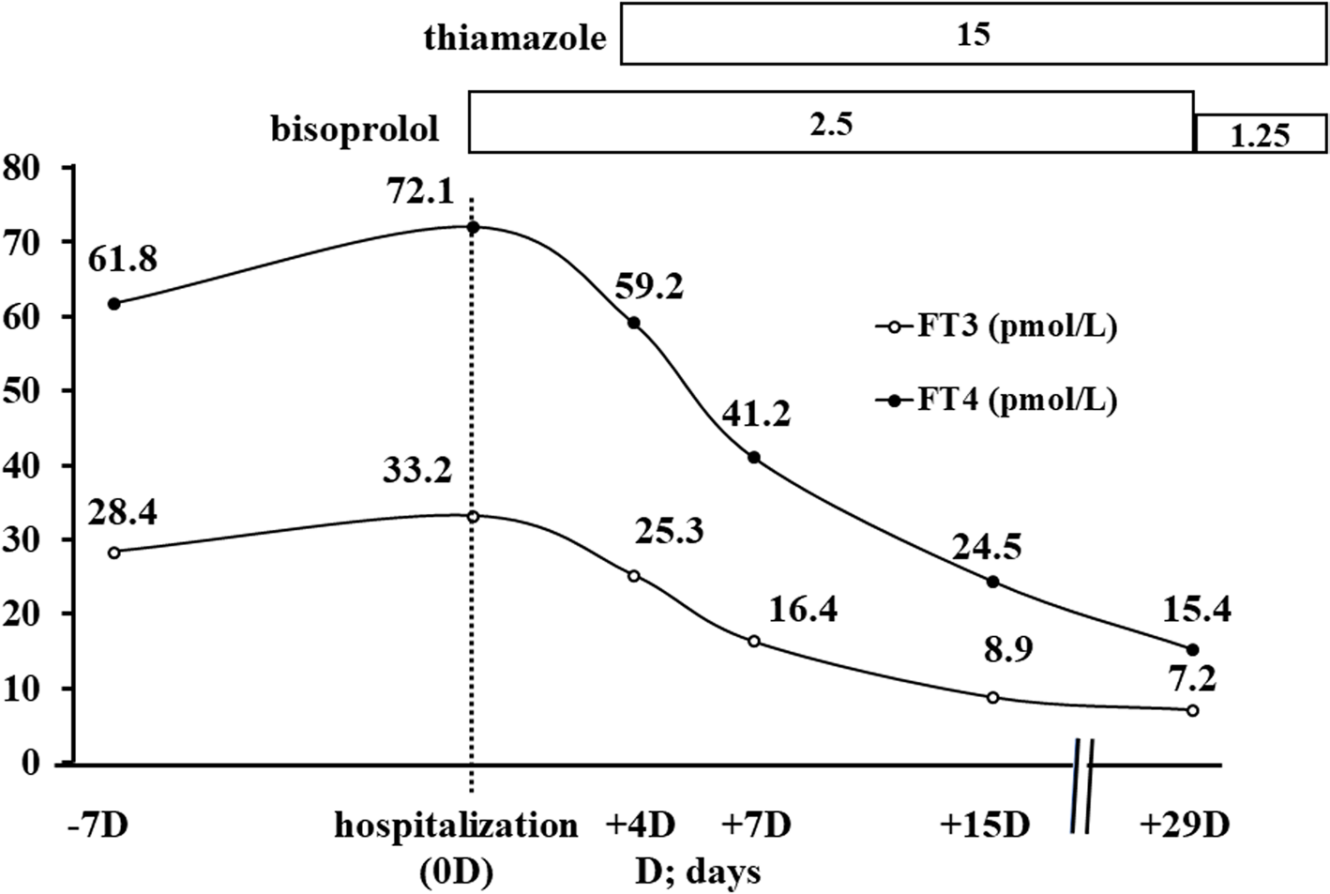
Clinical course. When the patient went to the emergency department because of palpitations associated with atrial fibrillation, her TSH was under the detection limit, and her thyroid hormone levels were elevated. After admission, she was absolutely rested and treatment with bisoprolol was initiated. After diagnosis with Graves’ disease, treatment with thiamazole was initiated and her symptoms and thyroid hormones improved dramatically

Thiamazole and bisoprolol were prescribed to correct her condition (Fig. 3). Soon after this treatment was initiated, her palpitations and fatigue were significantly relieved, and she was discharged from the hospital. After that, her thyroid hormone levels improved, and the bisoprolol dose was reduced. There were no side effects associated with the treatment.

## Discussion and conclusions

We reported a case of Graves’ disease following SARS-CoV-2 vaccination. Treatment with thiamazole was significantly effective for her condition.

Graves’ disease is an AITD marked by the presence of TRAb, with manifestations of diffuse goiter, thyrotoxicosis, and ophthalmopathy [1, 2]. The most common cause of hyperthyroidism is Graves’ disease. The causes of Graves’ disease are a loss of immunotolerance and the development of autoantibodies that stimulate thyroid follicular cells by binding to the TSH receptor. Our patient displayed symptoms due to excess thyroid hormones, such as palpitations due to atrial fibrillation, weight loss, fatigue, tremor, diarrhea, and menstrual disturbances. In addition, she demonstrated thyrotoxicosis with TSH levels under the detection limit and elevated levels of thyroid hormone, TRAb and TSAb positivity, and diffuse uptake in the thyroid gland on ^99m^Tc scintigraphy, so her symptoms were consistent with a diagnosis of Graves’ disease. She did not display diffuse goiter or ophthalmopathy. The low BMI found in our case was already present prior to the vaccination and is not part of the present discussion.

COVID-19 is caused by the novel beta-coronavirus SARS-CoV-2 [12, 13]. There are some reports of cases with Graves’ disease after COVID-19. The spike proteins covering SARS-CoV-2 bind to angiotensin-converting enzyme 2 (ACE2) receptors for its initial entry [14]. ACE2 expression levels are higher in the small intestine, testis, kidneys, heart, lung, adipose tissue, and thyroid [15]. SARS-CoV-2 may affect the thyroid via ACE2, the viral receptor, resulting in the onset of AITD, such as Graves’ disease [12, 13]. The cytokine storm associated with COVID-19 infection may also induce thyroid dysfunction.

An immunologic adjuvant is a substance that enhances the antigen-specific immune response without triggering one on its own [4, 5]. Adjuvants are commonly used to boost an immune response to treatments such as vaccination. ASIA was first coined in 2011 with the aim of codifying disorders characterized by innate and adaptive immune system dysregulation after adjuvant exposure [4]. Although the diagnostic criteria of ASIA are not clearly determined, the major signs of exposure to a preceding external stimulus (infection, vaccine, silicone, and adjuvant), ‘typical’ clinical manifestations, and improvement induced by removal of the inciting agent suggest a diagnosis of ASIA. In addition, the appearance of autoantibodies or antibodies directed at the suspected adjuvant is a minor diagnostic sign. Genetic predispositions for vaccine reactions such as ASIA remain uncertain [4]. ASIA includes various autoimmune diseases, such as collagen disease, blood disease, hepatic disease, neurological disease, and endocrine disease [5]. Most cases of AITD caused by ASIA are autoimmune subacute thyroiditis, which appear most often after exposure to human papillomavirus (HPV) vaccine, followed by influenza vaccine [8]. There are some reports of autoimmune Hashimoto's thyroiditis as an AITD; however, there are few reports of Graves' disease [6–8]. Our patient was exposed to the SARS-CoV-2 vaccine prior to clinical manifestations due to excess thyroid hormone and the appearance of thyroid antibodies of Graves’ disease. These findings suggested that the thyrotoxicosis in this case was due to Graves’ disease caused by ASIA.

To date, 11 different vaccines have been granted an emergency use listing by the World Health Organization and used worldwide, including mRNA, protein subunit, viral vector, and inactivated vaccines [9]. Similar to other vaccines, there are some reports of the development of autoimmune diseases after exposure to the SARS-CoV-2 vaccine, such as collagen disease, heart disease, blood disease, hepatic disease, and endocrine disease [10, 16, 17]. No predictive factors have been associated with SARS-CoV-2 vaccine-associated ASIA. Side effects after exposure to the SARS-CoV-2 vaccine were reported to each appropriate authority worldwide, and reports of Graves' disease caused by ASIA were included in these reports. The cases that had been published in journals at the time of submission are shown in Table 2. We identified a total of 62 cases of Graves’ disease following SARS-CoV-2 vaccination, excluding our case. All patients showed positive TRAb or thyroid-stimulating immunoglobulin, and TRAb-positive cases without thyrotoxicosis were excluded. The table was categorized according to the sales company of the vaccines. We identified 32 patients who received the Pfizer mRNA vaccine; [11, 18–36] six patients who received the Moderna mRNA vaccine; [20, 28, 31, 37–39] 12 patients who received mRNA vaccines from unknown companies; [40] ten patients who received the AstraZeneca vaccine, including those from the Serum Institute of India; [37, 41–44] and one patient each who received Johnson & Johnson and Sinovac vaccines [27, 38]. Characteristically, across all companies, most patients with Graves’ disease are relatively young women, which is consistent with Graves’ disease in general. In most cases, symptoms developed within several days after vaccination. Inconsistent with the common clinical signs of Graves’ disease, there were some patients without goiter. Almost all confirmed cases showed good treatment responsiveness. According to previous reports, patients and their immunological background were not related. There were few descriptions of ophthalmopathy and past history of COVID-19 infection, and we were not able to confirm these data. Comparing patients who received mRNA vaccines with those from other companies, we recognized frequent onset of symptoms after not only the first but also the second and later mRNA vaccinations.

**Table 2:** Reported cases of Graves’ disease after exposure to SARS-CoV-2 vaccination

	Our case	Pfizer (*n* = 32)	Moderna (*n* = 6)	Pfizer and Moderna (*n* = 50)	AstraZeneca (*n* = 10)	Johnson & Johnson (*n* = 1)	Sinovac (*n* = 1)
Vaccine Type	mRNA (Pfizer)	mRNA	mRNA	mRNA	Viral vector	Viral vector	Inactivated
Age, year	41	44.8 (22–74)	45.8 (36–63)	43.4 (22–74)	43.3 (19–70)	68	44
Male, n (%)	Female	9 (28)	2 (33)	12 (24)	3 (30)	Female	Female
Time of symptom onset after vaccination, day	14	18.5 (1–120)	16.3 (2–46)	18.7 (1–120)	13.2 (2–31)	32	7
Dose, n (%)	Second	First 21 (66) Second 10 (31) Third 1 (3)	First 3 (50) Second 3 (50)	First 29 (58) Second 20 (40) Third 1 (2)	First 9 (90) Second 1 (10)	First	First
Enlarged thyroid, n (%)	No	18/24 (75)	2/4 (50)	32/40 (80)	1/8 (13)	NA	NA
Relapse, n (%)	No	4 (13)	1/5 (20)	11/49 (22)	2 (20)	No	Yes
FT3, pmol/L	33.2	19.8 (7.8–44.1)	27.5 (n = 1)	21.3 (6.3–44.1)	30.7 (n = 1)	21.2	14.8
FT4, pmol/L	72.1	48.4 (21–108)	50.4 (20–77)	48.2 (14–108)	43.7 (29–61)	46.3	34.4
Positive Tg-Ab, n (%)	Yes	14/22 (64)	3/4 (75)	17/26 (65)	3/3 (100)	NA	Yes
Positive TPO-Ab, n (%)	Yes	17/26 (65)	5/5 (100)	22/31 (71)	7/8 (83)	Yes	Yes

*mRNA* messenger RNA, *FT3* free triiodothyronine, *FT4* free thyroxine, *Tg-Ab* thyroglobulin antibody, *TPO-Ab* thyroid peroxidase antibody, *NA* not available

The mRNA-based SARS-CoV-2 vaccine (Pfizer and Moderna) uses lipid nanoparticles to facilitate the transport of mRNA into cells and is widely used worldwide, including in our patient [45, 46]. The vaccine contains a number of excipients and lipids, one of which is based on polyethylene glycol (PEG), which may induce an immune response in specific individuals in rare cases [46]. In a viral vector vaccine (AstraZeneca and Johnson & Johnson), this role could be played by polysorbate 80; in an inactivated vaccine (Sinovac), this role could be played by aluminum salts [47, 48]. On the other hand, Vojdani et al. showed that many thyroid peroxidase (TPO) peptide sequences shared homology or similarity with sequences in various SARS-CoV-2 proteins. Furthermore, the SARS-CoV-2 spike protein, nucleoprotein, and membrane protein all cross-reacted with TPO. This suggests that cross-recognition between the modified SARS-CoV-2 spike protein encoded in the mRNA vaccine and thyroid target proteins may promote AITD [49, 50].

In summary, we reported a case of Graves’ disease after exposure to the SARS-CoV-2 vaccine. Treatment with thiamazole was significantly effective for her condition. This case report reinforces the potential correlation between ASIA affecting the thyroid and SARS-CoV-2 vaccination. The clinical course suggests that it is essential to consider the possibility of developing ASIA, such as Graves' disease, after exposure to the SARS-CoV-2 vaccine.

## Data Availability

The datasets used and/or analysed during the current study are available from the corresponding author on reasonable request.

## Abbreviations

AITD: Autoimmune thyroid disease
TSH: Thyroid stimulating hormone
TRAb: TSH receptor antibody
ASIA: Autoimmune/inflammatory syndrome induced by adjuvants
SARS-CoV-2: Severe acute respiratory syndrome coronavirus 2
mRNA: Messenger RNA
FT3: Free triiodothyronine
FT4: Free thyroxine
BMI: Body mass index
SpO_2_: Oxygen saturation
TSAb: Thyroid stimulating antibody
Tg-Ab: Thyroglobulin antibody
TPO-Ab: Thyroid peroxidase antibody
ACE2: Angiotensin-converting enzyme 2
HPV: Human papillomavirus
PEG: Polyethylene glycol
TPO: Thyroid peroxidase
